# Short-time prone posturing is well-tolerated and reduces the rate of unintentional retinal displacement in elderly patients operated on for retinal detachment

**DOI:** 10.1186/1471-2482-13-S2-S55

**Published:** 2013-10-08

**Authors:** Roberto dell'Omo, Francesco Semeraro, Germano Guerra, Marco Verolino, Mariapia Cinelli, Stefania Montagnani, Ciro Costagliola

**Affiliations:** 1 Department of Medicine and Health Sciences, University of Molise, Campobasso, Italy; 2 Eye Clinic, Department of Neurological and Vision Sciences, University of Brescia, Italy; 3 Unit of Ophthalmology, ASL N° 3 SUD, Boscoreale, Naples, Italy; 4 Department of Public Health, University of Naples "Federico II", Naples, Italy

## Abstract

**Purpose:**

To evaluate the feasibility, efficacy and safety of strict prone posturing taken for 2 hours after operation in preventing the occurrence of unintentional retinal displacement in elderly patients operated on for retinal detachment (RD).

**Methods:**

Twenty patients aged 60 or more with diagnosis of macula-off RD were asked to keep a strict face-down posturing for 2 hours after vitrectomy and 20% sulfur hexafluoride tamponade. IOP was measured immediately before and after surgery and after the 2-hour posturing. A questionnaire was administered to each patient to evaluate the rate of discomfort experienced because of the face-down posturing. Unintentional displacement of the retina was assessed by evaluating the presence of retinal vessel printings on fundus autofluorescence images taken 4 weeks after operation.

**Results:**

The 2-hour posturing was generally well-tolerated. A mild neck pain was the most common reported symptom. Only a few patients experienced moderate breath shortness while posturing and none had to break the posturing because of respiratory problems. Intraocular pressure (IOP) measured before operation (11.7 ± 2.6 mmHg) was significantly different from IOP recorded at the end of surgery (18.9 ± 4.9 mmHg) and from IOP measured 2 hours after surgery (16.8 ± 4.7 mmHg, P<0.05, Friedman test). IOPs measured immediately and 2 hours after surgery did not differ significantly. Fundus autofluorescence imaging showed RVPs in 7 eyes.

**Conclusions:**

This study shows that a 2-hour face-down posturing is effective in reducing the rate of retinal displacement in patients operated on for rhegmatogenous retinal detachment using vitrectomy and SF6 20%. A 2-hour face-down posturing is feasible for elderly patients and does not appear to cause unwanted, post-operative IOP raises.

## Introduction

**Figure 1:**
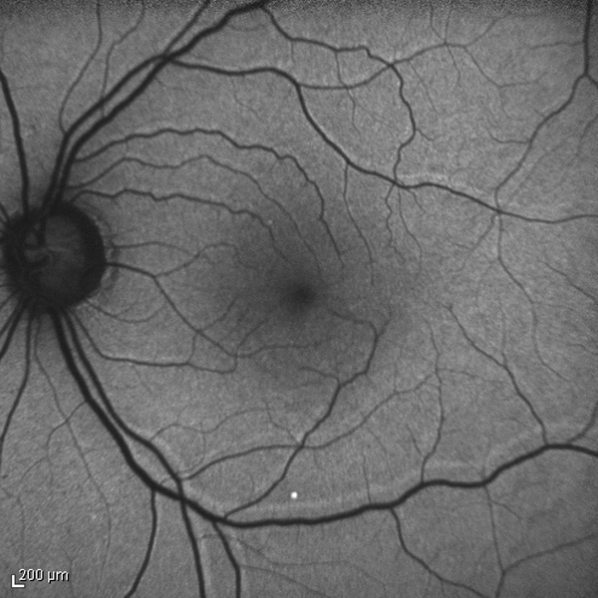
**Retinal vessel printings (RVPs) observed after retinal detachment repair**. RVPs are lines of increased autofluorescence running parallel to the retinal vessels they are related to. RVPs indicate the position of the retinal vessels before the occurrence of displacement.

Retinal detachment (RD) is most often the result of the retina becoming thinner and more brittle with age and pulling away from the underlying blood vessels; it can also be caused by a direct injury to the eye, but this is less common. Oxidative stress is a prominent feature of human eyes with primary RD, and is directly related to detachment severity [1]. In literature increased levels of NO pathway metabolites in the vitreous fluid of eyes with retinal detachment were described, which may reflect a possible role of NO in the pathogenesis of this disease [2]. Oxidative stress and elevated ROS (Reactive oxygen species) has been implicated in the mechanism of senescence and aging too. They are also involved in many diseases such cancer, diabetes, neurodegenerative, cardiovascular and other type of pathologies [3,4]. Overproduction of oxidant molecules is due to several stress agents such chemicals, drugs, pollutants, high-caloric diets and exercise [5]. Retinal vessel printings [6] are lines of increased autofluorescence secondary to displacement of retinal vessels from their original location (Figure 1). The displacement may occur as a consequence of RD [7] or because of tangential traction due to epiretinal membrane [8]. The displacement associated to RD may be favoured by the persistence of residual subretinal fluid at the end of the surgery and by injection of gas; its rate after standard vitrectomy for RD has been reported as high as 60% [7]. The displacement may lead to postoperative horizontal and vertical strabismus and to metamorphopsia, which may both impair the ability to perform the daily-life activity and prolong the recovery time after surgery. This is especially true for elderly people who can be affected by other ocular and systemic co-morbidities. It has been proposed that a strict prone position, taken immediately after operation, may prevent the retinal displacement [7]. However, a prolonged face-down posturing (FDP) can be demanding and difficult to adhere to by elderly patients that have co-morbidities, such as arthritis [9]. Complications of prone posturing include ulnar neuropathies [10,11] and acute intraocular pressure rises, possibly through an anterior shift of the iris-lens diaphragm, that promote both pupil block and anterior synechiae [12]. The aim of this study was to evaluate the feasibility, efficacy and safety of strict prone position taken for 2 hours after operation in preventing the occurrence of retinal displacement in a elderly group of patients undergone vitrectomy and gas for RD.

## Methods

All the patients aged 60 or more with diagnosis of rhegmatogenous RD involving the macula and attending the Ophthalmology Department at the University of Molise between January 7, 2012 and December 20, 2012 were asked to participate to this prospective study. The study was approved by the Institutional Review Board of the University of Molise and was in adherence to the tenets of the Declaration of Helsinki. Signed informed consent was obtained from all study subjects. Patients with previous history of ophthalmic surgery except non-complicated phacoemulsification, patients with glaucoma, age-related macular degeneration and ocular vascular diseases such as diabetic retinopathy and retinal vein occlusion were excluded. Before operation all the patients underwent a comprehensive ophthalmic examination including measurement of best-corrected visual acuity (BCVA) using early treatment diabetic retinopathy study (ETDRS) charts at 4 meters, anterior segment examination and dilated funduscopy. Visual acuity of counting fingers was converted to 1.4 logarithm of the minimum angle of resolution (logMAR), hand movements to 2.7 logMAR, and light perception to 3.7 logMAR. Before administering the subtenon anaesthesia, IOP was measured in the operating room with Tono-Pen XL applanation tonometer (Reichert technologies, Reichert Inc, Depew, USA). Twenty-five gauge pars plana vitrectomy was performed in all cases with the Constellation vitrectomy system (Alcon Labs, Fort Worth, TX). The surgical procedure involved separation and removal of the posterior hyaloid if not already detached, complete vitrectomy with shaving of the vitreous base and relief of all vitreous tractions on retinal tears. Perfluorocarbon liquid was not used. Particular care was paid to drain as much as possible subretinal fluid during air-fluid exchange procedure. Twenty percent of sulfur hexafluoride gas was used as internal tamponade in all cases. After removing the 25-gauge cannulas IOP was measured again with Tono Pen. Immediately after operation the patients were asked to maintain a strict face-down posturing for 2 hours with a five-minute interval after the first hour, independently from the location of the break/detachment. After the 2-hour posturing the patients underwent for a third time measurement of the IOP with Tono Pen. At the first post-operative visit, the day after surgery, a questionnaire was administered to each patient (Table 1) to evaluate the rate of discomfort experienced because of the face-down posturing. A score ranging from 0 (no discomfort) to 5 (severe discomfort) was assigned to each question and patients were invited to report the relative score according to their symptoms. A complete ophthalmologic examination was also carried out. Fundus autofluorescence (excitation wavelength at 488 nm and barrier filter at 500 nm), infra-red (IR) and red-free (RF) pictures (50 and 35 degrees) were obtained four weeks after operation when no residual gas was visible in the operated eye. Images were acquired with Spectralis HRA+OCT (Heidelberg Engineering, Heidelberg, Germany).

**Table 1:** Characteristics of the sample

Age (mean ± SD) years	64.3 (± 3.5)
Gender male/female	12/8

Pre-op logMAR VA (mean ± SD)	1.15 ± 0.88

Post-op logMAR VA (mean ± SD)	0.55 ± 0.30

Lens status Phakic/IOL	4/16

Duration of detachment (mean ± SD) days	6.25 ± 3.44

Quadrants detached	2.95 (± 0.75)

Location of the main break Superior/inferior quadrants	16/4

IOP pre-op	11.7 ± 2.6

IOP post-op	18.9 ± 4.9

IOP 2-hour post-op	16.8 ± 4.7

logMAR = logarithm of the minimum angle of resolution visual acuity,

IOP = intraocular pressure.

### Statistical analysis

Statistical analyses (Wilcoxon signed rank test and Friedman test) were performed using MedCalc version 11.5.1 (Med-Calc software, Mariakerke, Belgium). A P value < 0.05 was considered statistically significant.

## Results

Twenty patients (12 men and 8 women) were enrolled into the study. Mean age of the patients was 64.3 (± 3.5) years. Sixteen were pseudophakic, and 4 were phakic. Duration of detachment was 6.25 (± 3.44) days. Quadrants of retinal detachment were 2.95 (± 0.75).The main break was located in the superior quadrants in 16 eyes and in the inferior quadrants in 4 eyes. Preoperative mean (± standard deviation) logMAR visual acuity was 1.15 ± 0.88 (0.4-3.7) and improved to 0.55 ± 0.30 (0.1-1) postoperatively. The improvement was statistically significant (P < 0.0001; Wilcoxon signed rank test). IOP measured before operation (11.7 ± 2.6 mmHg) was significantly different from IOP recorded at the end of surgery (18.9 ± 4.9 mmHg) and from IOP measured 2 hours after surgery (16.8 ± 4.7 mmHg, P<0.05, Friedman test). However IOPs measured immediately and 2 hours after surgery did not differ significantly. The scores evaluating the level of discomfort secondary to face-down posturing are summarized in Table 2. In general all the patients managed to keep the face-down posturing for 2 hours without any major complaint. Fundus autofluorescence images recorded 4 weeks after operation showed RVPs in 7 eyes.

**Table 2:** Score of the discomfort secondary to face down position

	SCORE
**How much difficulty did you have keeping the prone position?**	**2.3 ± 1.67**

**Did you have breath shortness while posturing face-down?**	**1.6 ± 1.2**

**Did you suffer any neck or back pain while posturing face-down?**	**2.0 ± 1.5**

**Did you feel dizziness after the face-down posturing?**	**1.3 ± 0.6**


## Discussion

Ageing of the human vitreous is held to play a crucial role in the development of retinal tears subsequently leading to RD. Ageing of the human vitreous is characterized by gel liquefaction and the development of fluid-filled pockets which involves approximately one fifth of the total vitreous volume by the middle to late teenage years and at least 50% of the gel in most individuals older than 70 years. Factors that accelerate vitreous liquefaction and posterior vitreous detachment include inflammation, retinal vascular diseases, and cataract extraction. Rhegmatogenous retinal detachment occurs when liquefied vitreous fluid enters the subretinal space through a full-thickness retinal break. Population-based studies show that the annual incidence is about 10-15 in 100,000 with a prevalence of about 0.3% of the general population and a lifetime risk of 3% by the age of 85 [13]. The incidence increases to 17.9 per 100,000 if detachments after cataract extraction (a common risk factor) are included [14]. Outcomes after RD repair depend upon the length of time that the retina has been detached and whether the macula is involved: prognosis is related inversely to the degree of macular involvement and the length of time the retina has been off. In general, a patient presenting late with a 'macula-off' RD has a very bleak outlook and several weeks may be needed for the vision to improve after surgery (particularly if there is a gas bubble in situ). Even in cases in which retinal reattachment is achieved after a single operation, patients with macula-off detachment may complain of visual disurbances including poor visual acuity and metamorphopsia. RD cannot be addressed by utilizing endothelial progenitor cells-based therapy, which has otherwise been suggested to treat other forms of eye disease [15-17]. In particular metamorphopsia may result from unintentional retinal displacement which may be eleganty disclosed by fundus autofluorescence [6,7]. Since metamorphopsia may severely affect the performance of daily tasks, which can be already limited in elderly people, any effort should be done in order to limit its occurrence. In the present study we investigated if a strict 2-hour long face-down posturing was able to limit the occurrence of retinal displacement in patients undergone vitrectomy and gas to repair macula-off RD. In addition we wanted to evaluate the feasibility and safety of the posturing for elderly people. We noted that the rate of unintentional displacement as showed by FAF was much lower than that reported previously [7]. It suggests that a 2-hour posturing taken immediately after operation is effective in reducing the rate of unintentional displacement of the retina. It also appeared that a 2-hour posturing was generally well-tolerated by our sample of elderly patients and therefore feasible in the daily practice. A mild neck pain was the most common reported symptom. Only a few patients experienced moderate breath shortness while posturing and none had to break the posturing because of respiratory problems. In order to examine the safety of the procedure and its repercussions on IOP we took measurements of the ocular pressure three times (i.e. immediately before and immediately after operation and immediately after the face-down posturing). There was no significant difference between the IOPs recorded immediately after surgery and after the face-down posturing suggesting that the no-expansible mixture of SF6 20% and air is safe and does not induce IOP raise after short prone posturing. In conclusion, our results show that a 2-hour face-down posturing is effective in reducing the rate of retinal displacement in patients operated on for rhegmatogenous retinal detachment using vitrectomy and SF6 20%. A 2-hour face-down posturing is feasible for elderly patients and does not appear to cause unwanted, post-operative IOP raises.

## Authors' contributions

RDOM: conceived the study, analyzed and interpreted the data, drafted the manuscript. FS: conceived the study, critically revised the manuscript. GG: conceived the study, analyzed and interpreted the data, critically revised the manuscript. MV: conceived the study and critically revised the manuscript. MPC: critically revised the manuscript. SM: critically revised the manuscript. CC: conceived the study, analyzed and interpreted the data, drafted and critically revised the manuscript. All authors read and approved the final manuscript.

## Authors' information

RDOM: Assistant Professor of Ophthalmology at University of Molise. FS: Associate Professor of Ophthalmology at University of Brescia. GG: Assistant Professor of Anatomy at University of Molise. MV: Chief Staff Physician of Ophthalmology at Boscotrecase Hospital. MPC: Assistant Professor of Anatomy at University of Naples "Federico II". SM: Full Professor of Anatomy at University of Naples "Federico II". CC: Associate Professor of Ophthalmology at University of Molise.

## Declarations

Publication charges for this article were covered by research funds of the project Bando Faro 2012 - Finanziamenti per l'Avvio di Ricerche Originali, cofounded by the Compagnia di San Paolo and by the Polo per le Scienze e le Tecnologie per la Vita of the University Federico II in Naples.

This article has been published as part of *BMC Surgery *Volume 13 Supplement 2, 2013: Proceedings from the 26th National Congress of the Italian Society of Geriatric Surgery. The full contents of the supplement are available online at http://www.biomedcentral.com/bmcsurg/supplements/13/S2
